# Priming with Retinoic Acid, an Active Metabolite of Vitamin A, Increases Vitamin A Uptake in the Small Intestine of Neonatal Rats

**DOI:** 10.3390/nu13124275

**Published:** 2021-11-27

**Authors:** Yaqi Li, Cheng-Hsin Wei, J. Kalina Hodges, Michael H. Green, A. Catharine Ross

**Affiliations:** Department of Nutritional Sciences, The Pennsylvania State University, University Park, State College, PA 16802, USA; yxl277@psu.edu (Y.L.); ginawei420@gmail.com (C.-H.W.); hodges.466@psu.edu (J.K.H.); mhg@psu.edu (M.H.G.)

**Keywords:** vitamin A, retinoic acid, vitamin A supplementation, neonatal rats, intestine, tracer kinetics

## Abstract

Given that combined vitamin A (VA) and retinoic acid (RA) supplementation stimulated the intestinal uptake of plasma retinyl esters in neonatal rats, we administrated an RA dose as a pretreatment before VA supplementation to investigate the distinct effect of RA on intestinal VA kinetics. On postnatal days (P) 2 and 3, half of the pups received an oral dose of RA (RA group), while the remaining received canola oil as the control (CN). On P4, after receiving an oral dose of ^3^H-labeled VA, pups were euthanized at selected times (*n* = 4–6/treatment/time) and intestine was collected. In both CN and RA groups, intestinal VA mass increased dramatically after VA supplementation; however, RA-pretreated pups had relatively higher VA levels from 10 h and accumulated 30% more VA over the 30-h study. Labeled VA rapidly peaked in the intestine of CN pups and then declined from 13 h, while a continuous increase was observed in the RA group, with a second peak at 10 h and nearly twice the accumulation of ^3^H-labeled VA compared to CN. Our findings indicate that RA pretreatment may stimulate the influx of supplemental VA into the intestine, and the increased VA accumulation suggests a potential VA storage capacity in neonatal intestine.

## 1. Introduction

It is recognized that the first 24 months after birth is a critical period for postnatal development. Physiological changes occurring during this time affect the neonatal period and may have a long-term impact on later life stages [1,2,3]. A nutricious and well-balanced diet during this developmental period is of great importance for fulfilling the nutritional requirements of the neonate and for establishing a solid foundation for optimal health in future life [1,4,5,6,7]. However, limited information is currently available to formulate an accurate dietary recommendation of various nutrients for the early life stage [8].

Vitamin A (VA), as an essential micronutrient with a well-established role in a wide range of biological processes, including cell growth, tissue differentiation, and organogenesis in the prenatal period [9,10], as well as the postnatal development of various organs and systems, such as lung, adipose tissue, and neuronal and immune systems, across mammalian species [10,11,12,13]. In addition, retinoic acid (RA), as the most bioactive form of VA, has been found to fulfill many of the biological functions of VA. One of the key roles is in the regulation of immune functions [14], which is of unique importance during the childhood period given the fact that children are highly susceptible to common childhood infections, such as diarrheal, due to their developing and immature immune system, which is distributed across developing organs including the gut, liver, lungs, spleen, and skin. As reported by other research groups, VA deficiency is associated with a higher risk of gastrointestinal and respiratory morbidity and higher overall mortality in children younger than 59 months of age [15,16,17,18].

In order to eliminate VA deficiency and improve the health outcomes of children, the World Health Organization has implemented VA supplementation programs in low- and middle-income countries where the prevalence of VA deficiency is high [19]. Despite evidence demonstrating that VA supplementation reduced the risk of all-cause and diarrhea-related mortality in children from six months to five years of age [20,21], no conclusion was reached regarding the effect of VA supplementation for children younger than six months due to the inconsistency of findings, as, for instance, VA supplementation was found to be beneficial in some studies [22,23,24,25], but there was no statistical difference between VA supplementation and placebo groups in other studies [26,27]. To address this knowledge gap related to neonates, more evidence is required on how VA supplementation affects intestinal VA absorption, uptake, and utilization in this early life stage. 

Previously, using isotopic tracer methods and model-based compartmental analysis, we observed a dramatic increase in the intestinal uptake of chylomicron-derived retinyl esters (RE) and an increased exchange of retinol-binding protein-bound retinol (RBP-retinol) between intestine and plasma in response to supplementation with a combination of VA and RA (VARA), compared to placebo control, in a neonatal rat model [28]. While VARA supplementation provided the potential benefit of preferentially allocating VA to intestine, as well as lung [28], whether an RA pretreatment, as a priming treatment, would magnify or attenuate this beneficial effect of a subsequent VA supplement is unknown. Therefore, in the current study, to examine the distinct effect of RA priming on intestinal VA metabolism and kinetics, we provided RA as a pretreatment prior to VA supplementation in neonatal rats, compared to oil-pretreated rats that also received VA supplementation as the control (CN). We hypothesized that there could be either one of two outcomes: First, RA might serve as a signal of VA adequacy to the neonatal body, activating a feedback regulatory process to limit intestinal uptake of a subsequent VA dose, similar to the effect of RA on ß-carotene absorption [29,30]. Or, conversely, RA might potentially function as a feed-forward “priming” signal, informing the body that more VA is likely “on its way,” and thus RA priming might exhibit a synergetic effect on VA absorption, similar to our observation for the neonatal lung in previous kinetic studies [28,31]. By pretreating neonates with RA prior to providing a VA supplemental dose, and subsequently determining the kinetics of VA mass and uptake of ^3^H-labeled-retinol contained in the supplement, we hoped to be able to distinguish between these two potential effects of RA on the intestine.

## 2. Materials and Methods

### 2.1. Animals and Diets

Pregnant female Sprague-Dawley rats were fed a purified VA-marginal diet (Research Diets, New Brunswick, NJ, USA), formulated based on the AIN93G diet to contain 0.35 mg retinol equivalents/kg diet [32]. Rats had continuous access to food and water and were housed in an environmentally-controlled animal facility with a 12-h light/dark cycle. All procedures involving animals were approved by the Institutional Animal and Care and Use Committee of The Pennsylvania State University.

### 2.2. Oral Dose Preparation

All-trans RA and VA (in the form of all-trans retinyl palmitate) were purchased from Sigma-Aldrich (St. Louis, MO, USA). Based on the WHO recommendation for VA supplements (50,000 IU) provided to human infants to reduce morbidity and mortality and on previous studies [19,28], we prepared the oral dose for the RA pretreatment as 0.6 μg RA/g body weight and the VA supplementation as 6 μg retinol/g body weight for the neonatal rats. For adding the radioactive label to the VA supplement, briefly, a known amount of 11, 12-^3^H-retinol (Perkin-Elmer, Waltham, MA, USA) was added to the VA supplement, making the final isotope concentration a ~0.8 μCi/μL dose [28]. On the day of dose administration, replicate aliquots of dose were analyzed by liquid scintillation spectrometry to determine and calculate the radioactivity level per dose administered.

### 2.3. Kinetic Studies

Pups were randomized within litters into two groups after birth. The entire study utilized 120 neonates randomized from 13 litters, thus litter effects were balanced in this study. On postnatal days (P) 2 and 3, one group (RA group) received the oral RA dose as a pretreatment, while the other group received canola oil (CN group). On P4, the VA dose containing 11,12-^3^H-retinol as the tracer was administered to all pups. Each dose was delivered directly into the mouth of the pup using a positive-displacement micropipette (Gilson, Middleton, WI, USA), after which a small piece of paper towel (chip) was used to wipe away any remaining dose from the muzzle; this chip, as well as the dosing pipette tip, were extracted and counted for correction of actual dose delivered to each neonate. 

Pups were returned to their dam immediately after dose administration, and all of them had access to mother’s milk throughout the study. At each of 12 pre-selected times after dose administration (25 min, 45 min, 1 h-15 min, 2 h, 3 h, 4.5 h, 6 h, 8 h, 10 h, 13 h, 20 h, 30 h), pups (*n* = 4–6/treatment/time) were euthanized with isoflurane (Phoenix Pharmaceutical, Burlingame, CA, USA). Intestines were collected and flushed with PBS to remove the contents, then stored at −80 °C until analysis.

### 2.4. VA Mass Quantification

For analysis, the entire intestine was divided into four segments: duodenum, jejunum, ileum, and colon [33], then each tissue segment was analyzed separately for its VA mass. Using a modification of a previous method [34], tissues were weighed and homogenized and VA was extracted into hexane (4 mL) without saponification; then a 0.5-mL aliquot of the hexane extract was used to measure VA mass by ultra-performance liquid chromatography (UPLC; Waters, Milford, MA, USA).

### 2.5. ^3^H Tracer Determination

A 0.5 mL aliquot of the above-described hexane extract was evaporated to dryness in a water bath under a gentle flow of nitrogen, then used for determination of ^3^H tracer by liquid scintillation spectrometry (Beckman Coulter, Brea, CA, USA) using Scintiverse (Fisher Chemical, Waltham, MA, USA) as scintillation fluid, as described earlier [35]. Samples were counted to a 2-sigma error of 1% or for a maximum of 60 min. 

To further identify the form of VA in intestine, we separated retinyl esters (RE) and retinol from the hexane extract using alumina column chromatography [36]. Briefly, the hexane extract was loaded onto the column, and then the column was sequentially eluted with 3% diethyl ether and 50% diethyl ether to obtain the retinyl ester- and retinol fractions, respectively. Then, ^3^H content in these fractions was determined as mentioned above.

### 2.6. Kinetic Data Calculations

The fraction of the administrated dose (FD) in the form of total retinoid, RE, and retinol in each segment of the intestine for each pup was calculated as radioactivity (dpm) in each measured tissue segment divided by total dpm administrated. The total radioactivity administered to each rat was calculated based on the measured dpm/μL in the oral dose × given volume; a correction was applied for each pup based on the radioactivity remaining in the chip and tip used for dosing. The mean FD at each sampling time for each of the two groups was calculated to generate tracer response curves for each treatment. Areas under the curve (AUC) per treatment were calculated using GraphPad Prism 9.0.

### 2.7. Statistical Analysis

Data for VA mass and tracer responses at each time are reported as mean ± SEM. Differences between groups were determined by 2-way ANOVA with multiple comparison test in GraphPad Prism 9.0. A *p* value < 0.05 was considered statistically significant. 

## 3. Results

### 3.1. RA Pretreatment Stimulated VA Accumulation in the Intestine of Neonatal Rats

**Figure 1:**
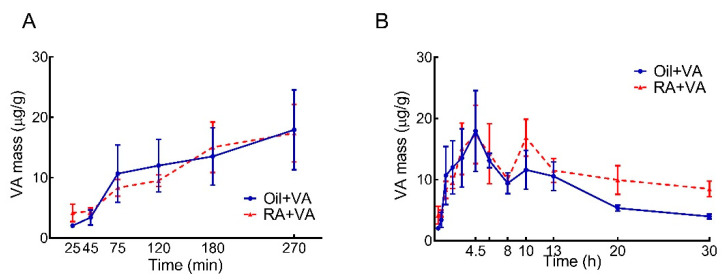
Intestinal VA concentration vs. time after oral administration of VA supplement in neonatal rats: (**A**) During the first absorptive phase; (**B**) Over the entire study. Data are presented as means ± SEM, *n* = 4–6 pups/time/group.

Changes in VA concentration over time were measured in neonatal duodenum, jejunum, ileum, and colon, and the VA content for the whole intestine was calculated as the sum of each of these segments, as shown in Figure 1. VA concentration rapidly and continuously increased after oral VA supplementation. The rate and scale of increase were comparable in the RA-pretreated and CN groups (Figure 1A). Concentrations reached their first peak at 4.5 h (Figure 1B), which were more than 7 times higher than the concentrations at baseline (25 min after dosing): 17.9 vs. 2.04 μg/g for the RA group and 17.4 vs. 4.18 μg/g for the CN group. In both groups, the concentrations declined from the peak at 4.5 h until 8 h, but then, interestingly, increased again, reaching a second peak at 10 h. From 10 h onwards, the RA-pretreated pups showed a tendency of retaining more VA in the intestine than the oil-pretreated CN pups, as shown in Figure 1B, such that the RA group maintained a relatively higher VA concentration during these later hours, even though the absolute VA concentration gradually decreased after the second peak in both groups. Despite the observed differences in kinetic patterns between two groups, there was no statistically significant difference between the RA primed and CN group (*p* = 0.26) for the entire study period. 

To examine the possibility that there might be differences in the integrated accumulation of VA mass in the two groups, which might represent a physiologically meaningful effect of RA pretreatment, we calculated the AUC for each group, based on VA concentration over time. Over the 30-h kinetic study, the AUC values (Table 1) indicated there was a 30% greater accumulated VA mass in the RA-primed pups compared to CN pups (332 vs. 251 μg/g × h). Notably, during the first wave of VA absorption (defined as 0–8 h after VA supplementation), the AUC values were almost identical for RA-pretreated and CN pups. The 30% greater observed increase in AUC in the RA-primed group was primarily attributable to the second wave of VA uptake, which happened after 8 h, and resulted in an elevated intestinal VA concentration in RA-primed pups that lasted to the end of the kinetic study.

### 3.2. RA Stimulated the Uptake of Newly Ingested ^3^H Labeled VA 

**Figure 2:**
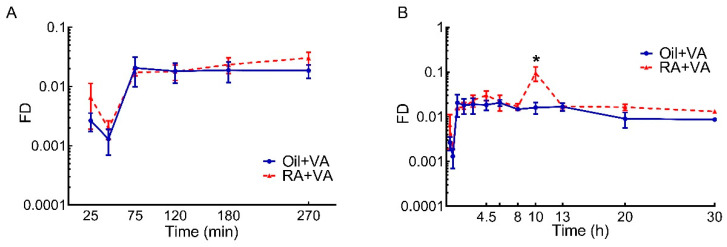
Fraction of ingested dose (FD) in the intestine vs. time after administration of ^3^H-labeled VA supplement in neonatal rats: (**A**) During the first absorptive phase; (**B**) Over the entire study. Data are presented as means ± SEM, *n* = 4–6 pups/time/group. * indicates statistically significant difference between groups, *p* < 0.05.

As for VA mass, the tracer response for neonatal whole intestine were calculated from the individually measured ^3^H content in each segment. Tracer response data are shown in Figure 2. The fraction of ingested dose present as total retinoids in the intestine increased dramatically within 75 min after ^3^H-retinol administration in both RA-pretreated and CN pups (Figure 2A). After this time, tracer present in total retinoids in the CN group remained nearly steady until 13 h after dose administration, then gradually decreased (Figure 2B). In contrast in RA-primed pups, after the first peak at 75 min, the total retinoids in the intestine exhibited a slight but continuous increase, reaching a second peak at 4.5 h after dose administration. After a drop, the curve showed a robust increase, reaching another peak at 10 h (Figure 2B), which was significantly higher compared to the CN group at 10 h (*p* < 0.0001). This peak was short lived, and at 13 h, the tracer amount detected in total retinoids had returned to its pre-peak value. From that point, a slight but gradual decrease was observed (Figure 2B). An overall treatment effect of RA pretreatment on total retinoids kinetics in the intestine of these neonatal rats over the 30-h time course was detected (*p* = 0.0136). In addition, the AUC values calculated from tracer kinetic curves over the 30-h study were nearly double in RA-pretreated pups compared to CN pups (0.702 vs. 0.39, Table 2). More specifically, during the first 8 h after dosing, newly ingested tracer rapidly accumulated in the neonatal intestine, regardless of the pretreatment, as suggested by the close AUC values in the two groups during this time (0.156 vs. 0.132). However, after that, whereas no further increase in tracer level was observed in oil-pretreated CN pups, the RA-primed pups exhibited another wave of tracer increase, resulting in higher tracer accumulation in later hours that lasted to the study’s conclusion.

### 3.3. VA Distribution in Duodenum, Jejunum, Ileum and Colon

**Figure 3:**
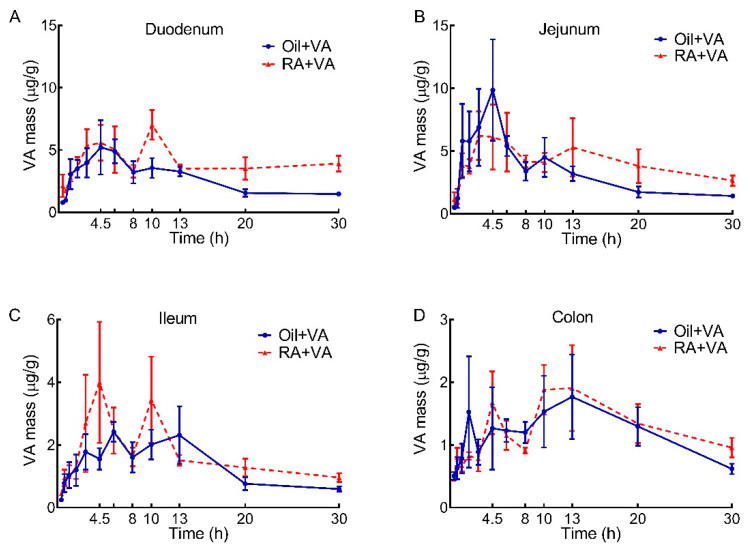
Regional VA distribution vs. time after oral administration of VA supplement in neonatal rats: (**A**) Duodenum; (**B**) Jejunum; (**C**) Ileum; (**D**) Colon. Data are presented as means ± SEM, *n* = 4–6 pups/time/group.

To determine if the effects of RA were observed along the entire intestine, VA masses in duodenum, jejunum, ileum, and colon were calculated for each segment (Figure 3A–D). A rapid increase in VA concentration in the early hours after oral VA supplementation was observed in all segments, with the majority proportion of VA present in duodenum and jejunum. VA concentration in duodenum and jejunum each reached their first peak at 4.5 h after dose administration, with comparable peak values in the CN and RA groups (Figure 3A,B). However, as the VA concentration in the duodenum and jejunum of CN pups began to decline after 4.5 h, RA-pretreated pups exhibited a second peak at 10 h and 13 h in duodenum and jejunum, respectively (Figure 3A,B); these segments were the primary contributors of the later peak that was observed in the whole intestine (Figure 1B). After the second peak, VA concentration remained elevated in the duodenum and jejunum of RA-pretreated pups compared to controls; differences between groups were significant for duodenum (*p* = 0.01) but not for jejunum. In addition, as shown in Table 3, the AUC values over the 30-h study indicated that VA mass accumulation in the duodenum of RA-pretreated pups was 50% greater than in CN pups (121 vs. 78.9 μg/g × h). This pretreatment effect was also observed for the jejunum, as supported by a nearly 30% higher VA mass in the RA-primed group compared to the CN group (124 vs. 97.9 μg/g × h). In comparison, VA concentration in ileum and colon fluctuated within a relatively small range, showing a first peak at 4.5–6 h and then reaching a second peak between 10–13 h, followed by a decline. The AUC for these segments indicated a slightly higher VA mass accumulation in the ileum of RA-pretreated pups (50.9 vs. 40.0 μg/g × h), whereas colon was the site with the lowest amount of VA mass accumulation, and there was no difference between groups (39.3 vs. 36.5 μg/g × h).

### 3.4. RA Pretreatment Stimulated the Uptake of ^3^H-Labeled VA in Duodenum, Jejunum, Ileum and Colon 

**Figure 4:**
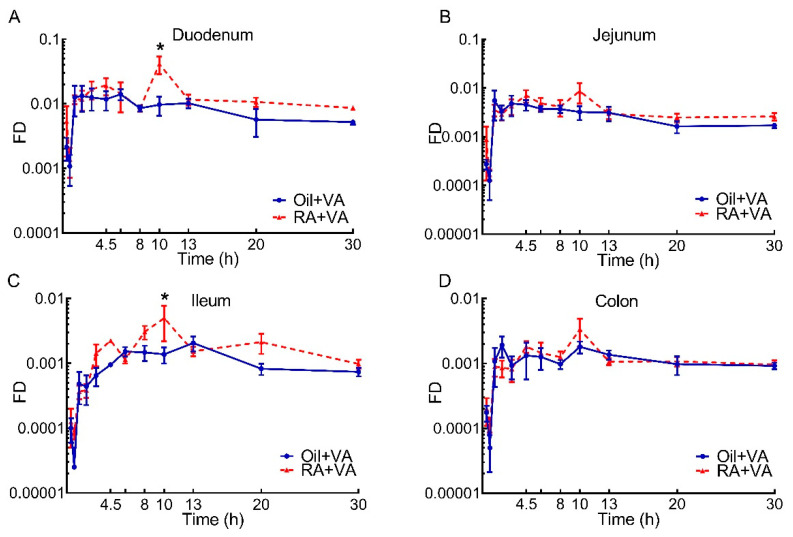
Regional distribution of fraction of ingested dose (FD) vs. time after administration of ^3^H-labeled VA supplement in neonatal rats: (**A**) Duodenum; (**B**) Jejunum; (**C**) Ileum; (**D**) Colon. Data are presented as means ± SEM, *n* = 4–6 pups/time/group. * indicates statistically significant difference between groups, *p* < 0.05.

Changes in the fraction of the ingested ^3^H dose in total retinoids in duodenum, jejunum, ileum, and colon are shown in Figure 4. Duodenum and jejunum took up most of the newly ingested dose compared to the two lower intestinal sections, with a greater portion in duodenum, independent of pups’ pretreatment. For oil-pretreated CN pups, the fraction of dose in total retinoids increased dramatically in the first 2 h after dosing and peaked between 75 min and 2 h, after which time retinoids in the duodenum and jejunum remained nearly constant for the following 4–6 h, then gradually decreased (Figure 4A,B). In contrast to the duodenum and jejunum, total retinoids in the ileum and colon, after the period of rapid increase in the first 2 h, fluctuated but continuously increased until 10–13 h after dose administration, then started to decrease (Figure 4C,D). The kinetic behavior of tracer in the four intestinal segments was similar in the RA-pretreated pups, with a rapid and dramatic increase in the first 75 min after dose administration; then, after approximately 2 h, total retinoids climbed again and reached a second and higher peak at 10 h, with different peak values in each segment (duodenum > jejunum > ileum > colon). After 10 h, total retinoids in all segments gradually decreased until the end of the study (Figure 4). The effect of RA pretreatment was significant on total retinoid kinetics in the duodenum (*p* = 0.0156) and ileum (*p* = 0.0223), with the fraction of dose in retinoids at 10 h postdosing significantly higher in RA-pretreated pups in both of these segments as compared to those in CN pups (*p* < 0.0001 and *p* = 0.0048, respectively). A relatively higher proportional increase in AUC with RA-pretreatment was also observed in duodenum and ileum (Table 4).

### 3.5. A Higher Proportion of Newly Ingested ^3^H Labeled VA Was Present in RE Than Retinol

**Figure 5:**
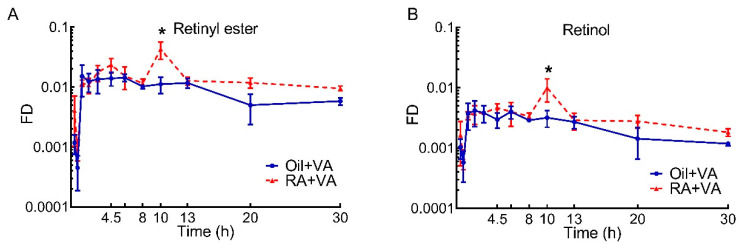
Fraction of ingested dose (FD) present as retinyl ester and retinol vs. time after administration of ^3^H-labeled VA supplement in neonatal rats: (**A**) RE; (**B**) Retinol. Data are presented as means ± SEM, *n* = 4–6 pups/time/group. * indicates statistically significant difference between groups, *p* < 0.05.

To further distinguish VA tracer kinetics in neonatal intestine in terms of the major forms of retinoid, we separated labeled VA into RE and retinol. As shown in Figure 5, the kinetic patterns for these two forms were almost identical. Newly ingested ^3^H tracer rapidly appeared in both RE and retinol, peaking in the intestine at 75 min at comparable level in RA and CN pups. While the elevated tracer level in both RE and retinol remained stable in CN pups for the next ~12 h, tracer in RE and retinol spiked at 10 h in the RA group, reaching significantly higher values than that in the CN group for both VA forms (*p* = 0.0002 for RE and *p* = 0.0108 for retinol). Then, RE and retinol in both groups gradually declined. Of note, the fraction of the ingested dose present in RE at different times was always 2–4 times higher than that in retinol, making RE the primary form of VA in pup intestine at all times. This observation was confirmed by the AUC results (Table 5): independent of pretreatment, RE was ~4 times higher than retinol (0.263 vs. 0.067 in oil-pretreated CN pups and 0.444 vs. 0.103 in RA-pretreated pups). Moreover, RA pretreatment stimulated the enrichment of the newly ingested tracer in RE to a greater extent than retinol. As a result, an overall RA pretreatment effect was detected for RE kinetics after VA dosing (*p* = 0.0163), whereas no significant effect was found for retinol kinetics. Tracer response curves for RE and retinol in each segment are included in Figures S1 and S2, which show kinetic patterns similar to the results illustrated in Figure 4.

## 4. Discussion

In the current study, female Sprague-Dawley rats were maintained on a VA marginal diet during pregnancy and lactation, which effectively restricted the maternal transfer of VA to the newborns; thus, these rat pups may be a good model for infants who are at risk of VA deficiency, such as those in low-income countries. While a previous study revealed that VARA supplementation stimulated the intestinal uptake of newly ingested VA [28], here, instead of giving RA and VA as a combined dose, the pups were pretreated with RA before the routine VA supplementation, and compared to pups that did not receive RA but did receive the same VA supplementation. In this manner, we tested whether RA pretreatment would significantly affect VA kinetics in intestine. We collected kinetic data over a 30-h study to determine the dynamic changes in VA mass and we characterized the tracer response in different VA forms in duodenum, jejunum, ileum, and colon, and in terms of RE compared to retinol.

From the VA mass quantification results for the total intestine (Figure 1A,B), we found that VA supplementation increased VA content in the intestine of neonatal rats within the first 4.5 h after dosing, regardless of the pretreatment. However, in comparison to CN pups, the 30% increase in AUC integrated over the 30-h study period in RA-pretreated pups (Table 1), which is primarily attributable to the observed second peak at 10 h and the generally higher VA level throughout the following hours, suggested a potential effect of RA on stimulating the uptake, retention, or even the recycling of VA in the intestine of neonatal rats. When we further examined the individual intestinal segments (Figure 3), the duodenum and jejunum, as expected, contributed more than 70% of the VA mass detected in the whole intestine (Table 3), which is in agreement with the common understanding that duodenum and jejunum are the major sites for VA absorption in the gastrointestinal (GI) tract [37,38]. Furthermore, as the primary contributor to the second VA peak, the observed VA mass change in duodenum suggested a potential stimulatory effect of RA pretreatment. Although this effect did not reach statistical significance in jejunum, both jejunum and duodenum retained a relatively higher VA concentration in later hours, suggesting that the observed positive regulatory effect of RA on intestinal VA uptake acted primarily on these major VA absorption sites and that RA stimulated another round of VA absorption in later hours after VA supplementation.

When comparing the dose-response curves for ^3^H-tracer from the two pretreatment groups (Figure 2), the stimulatory effect of RA pretreatment on the intestinal uptake of newly ingested labeled VA became more obvious, suggesting that the lack of significance in the RA pretreatment effect on VA mass may be masked by the preexisting VA in the intestine, which could vary depending on when the neonates last suckled, and thus contributing to greater variability than was observed for the ^3^H tracer data. It may indicate that the tracer kinetic curves better represent the real-time response in VA kinetics for newly absorbed VA after dosing, compared to total VA mass dynamics. Despite the parallel increases in intestinal tracer in the initial hours after VA supplementation that was observed for the two groups (Figure 2A), the capacity for taking up additional VA at later times appears to have been activated in RA-pretreated pups, leading to a significantly higher tracer level at 10 h in retinoids, RE, and retinol (Figure 2 and Figure 5) and a later overall increase in accumulated tracer (Table 2 and Table 5). It has been reported that RA can increase the expression of cellular retinol-binding protein-II (CRBP-II), considered to be the major intracellular carrier of retinol during intestinal VA absorption [39], and increase the uptake of retinol in human intestinal Caco-2 cells [40]. If similar mechanisms exist in the intestine of neonatal rats, it could be a clue to explain the observed stimulatory effect of RA pretreatment on the uptake of newly ingested VA. It is noteworthy that, while RA stimulated the enrichment of tracer in both RE and retinol components in RA-pretreated pups (Figure 5), a proportionally higher increase was found in RE, suggesting that RA pretreatment may promote the uptake of newly synthesized RE from chylomicrons formed after VA dosing through the serosal blood supply, as was previously suggested to occur in intestine and lung of neonatal rats supplemented with VARA (VA and RA simultaneously) [28,31], as well as in rats with low VA status that received RA in their diet [41]. Alternatively, it may be that RA pretreatment preferentially stimulates the synthesis of RE, the storage form of VA and the form incorporated into chylomicrons, in the intestine of neonatal rats, in line with the observations of Senoo et al. [42], which indicated the potential capacity of the intestine as a VA storage organ. In this case, it would allow VA to be readily available for local functional use given the importance of adequate VA in the functioning of the intestinal immune system, which may be especially important at this early life stage when mucosal immunity is still developing [15,16,17,43,44,45,46]. Additionally, the nearly flattened terminal slope of the dose-response curve in the RA-primed group indicated a relatively small disposal/utilization rate for VA and may also suggest active recycling of RBP-bound retinol between intestine and the plasma pool, which was previously demonstrated between plasma and extravascular tissues. Moreover, in data not shown, we did not observe differences in liver tracer kinetics between RA-pretreated and CN neonates, suggesting the uniqueness of the intestine compared to liver in response to RA pretreatment. Data collected in both liver and intestine will be applied in future compartmental modeling analysis, and all the possibilities mentioned above should be examined [47,48].

Notably, because we obtained tracer kinetic data for 4 intestinal segments (duodenum, jejunum, ileum, and colon), we were able to examine and identify the potential similarities and differences in VA kinetics in various parts of the GI tract in these neonatal rats. Interestingly, within each pretreatment group, the overall kinetic pattern in the various segments was very similar for both RE and retinol tracer (Figure 4, Figures S1 and S2). However, from the quantitative point of view, duodenum and jejunum contained the majority of labeled VA compared to ileum and colon, confirming our finding that most of the total intestinal VA mass was present in duodenum and jejunum and further supporting the idea that upper intestine took the primary responsibility for uptake of newly ingested VA (Figure 3 and Figure 4). Separation of total retinoids into RE and retinol revealed that RE was the predominant form of retinoids in the duodenum and jejunum, while not much difference was observed in the ratio of RE and retinol in ileum and colon (Figures S1 and S2). These results suggest that duodenum and jejunum, the upper segments of the small intestine, were the sites for VA uptake and had the potential to store VA.

Our study explored the impact of RA, a bioactive VA metabolite, on the kinetics and metabolism of VA in neonatal intestine; the results indicate a positive regulatory effect of RA on the absorption of a subsequently-administered VA supplement. Based on our experimental design, with frequent sampling and detailed analysis of duodenum, jejunum, ileum, and colon, we identified the specific time, beginning at ~10 h after VA dosing, that reflected a unique kinetic difference due to RA pretreatment. We also found a similar VA kinetic pattern in different intestinal segments in response to RA pretreatment. Although our analyses required the use of all the sample material, and therefore additional samples are not available from this study to further investigate changes at the molecular level, changes such as differences in the expression of VA metabolism-related genes will be of interest in the future. The data presented here help to generate new hypotheses regarding whether RA may up-regulate processes that enhance the absorption, retention, or even serosal uptake of VA after its initial mucosal absorption and entry into the body, which could be further tested in the future using compartmental modeling.

## 5. Conclusions

Our findings identified the effect of RA pretreatment in stimulating the uptake of a VA supplement in the small intestine of neonatal rats: RA pretreatment induced increased intestinal VA accumulation, preferentially in the form of RE, which suggested the potential VA storage capacity of intestine, primarily in duodenum and jejunum, in neonatal rats.

## Figures and Tables

**Table 1:** Area under the curve (AUC) in RA-pretreated pups and CN pups, indicating higher intestinal VA mass accumulation in RA-primed pups in the later hours after VA supplementation.

	Area under the Curve, μg/g × h				
Selected Time Interval, h	0–8	8–13	13–20	20–30	Total (0–30)
Oil + VA	95.1	54.2	55.5	46.5	251
RA + VA	95.8	69.3	74.9	92.0	332

**Table 2:** Area under the curve indicated higher intestinal ^3^H-labeled VA accumulation in RA-pretreated pups at later hours after VA supplementation.

	Area under the Curve				
Selected Time Interval, h	0–8	8–13	13–20	20–30	Total (0–30)
Oil + VA	0.132	0.080	0.090	0.088	0.39
RA + VA	0.156	0.282	0.117	0.147	0.702

**Table 3:** VA mass accumulation in each intestinal segment after VA supplementation in oil-pretreated and RA-pretreated neonates.

	Area under the Curve, μg/g × h			
Intestinal Segment	Duodenum	Jejunum	Ileum	Colon
Oil + VA	78.9	97.9	40.0	36.5
RA + VA	121	124	50.9	39.3

**Table 4:** ^3^H labeled VA accumulation in each intestinal segment after VA supplementation with and without RA pretreatment.

	Area under the Curve			
Intestinal Segment	Duodenum	Jejunum	Ileum	Colon
Oil + VA	0.244	0.0792	0.0330	0.0339
RA + VA	0.406	0.108	0.0569	0.0379

**Table 5:** ^3^H-labeled VA present as RE and retinol in neonatal intestine after VA supplementation.

		Area under the Curve				
Selected Time Interval, h		0–8	8–13	13–20	20–30	Total (0–30)
Retinyl ester	Oil + VA	0.094	0.056	0.059	0.054	0.263
	RA + VA	0.113	0.138	0.086	0.107	0.444
Retinol	Oil + VA	0.025	0.015	0.014	0.013	0.067
	RA + VA	0.028	0.032	0.020	0.023	0.103

## Data Availability

The data that support the findings of this study are available from the corresponding author upon reasonable request.

## Supplementary Materials

The following are available online at https://www.mdpi.com/article/10.3390/nu13124275/s1, Figure S1: Fraction of ingested dose existing in retinyl ester in different segments of intestine vs. time after administration of ^3^H-labeled VA supplement in neonatal rats, Figure S2: Fraction of ingested dose existing in retinol in different segments of intestine vs. time after administration of ^3^H-labeled VA supplement in neonatal rats.
