# Supplementary material for: Priming with Retinoic Acid, an Active Metabolite of Vitamin A, Increases Vitamin A Uptake in the Small Intestine of Neonatal Rats
Source: Nutrients. 2021 Nov 27;13(12):4275. doi: 10.3390/nu13124275 (PMC8703606; doi:10.3390/nu13124275)
Supplement: Supplementary file 1 [file nutrients-13-04275-s001.zip › nutrients-1481221-supplementary.pdf]

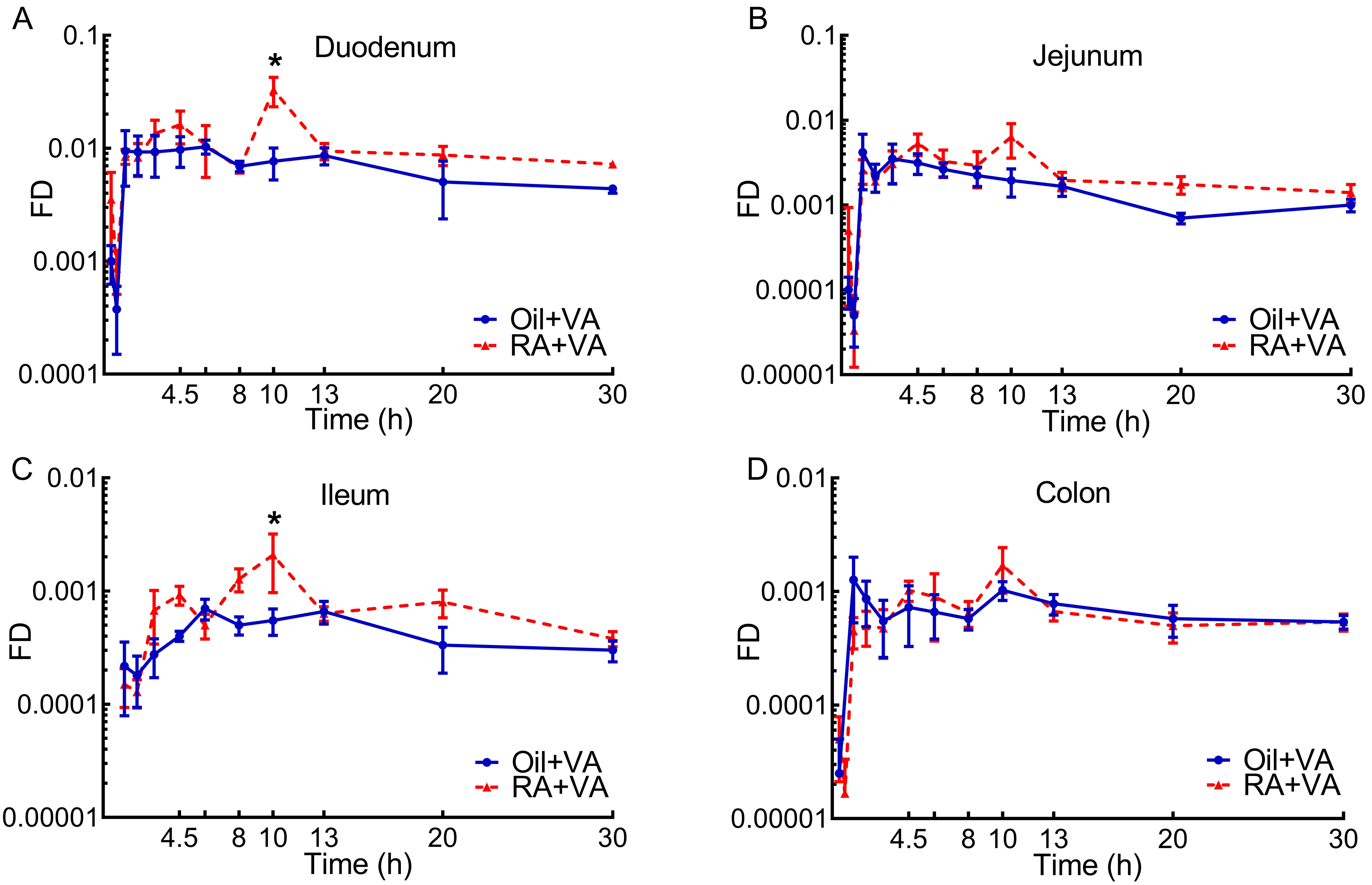

Figure S1. Fraction of ingested dose existing in retinyl ester in different segments of intestine vs. time after administration of ^{3}H-labeled VA supplement in neonatal rats. (A) Duodenum, (B) Jejunum, (C) Ileum, (D) Colon. Data are presented as means ± SEM, *n*=4-6 pups/time/group. * indicates statistically significant difference between groups, $P < 0.05$.

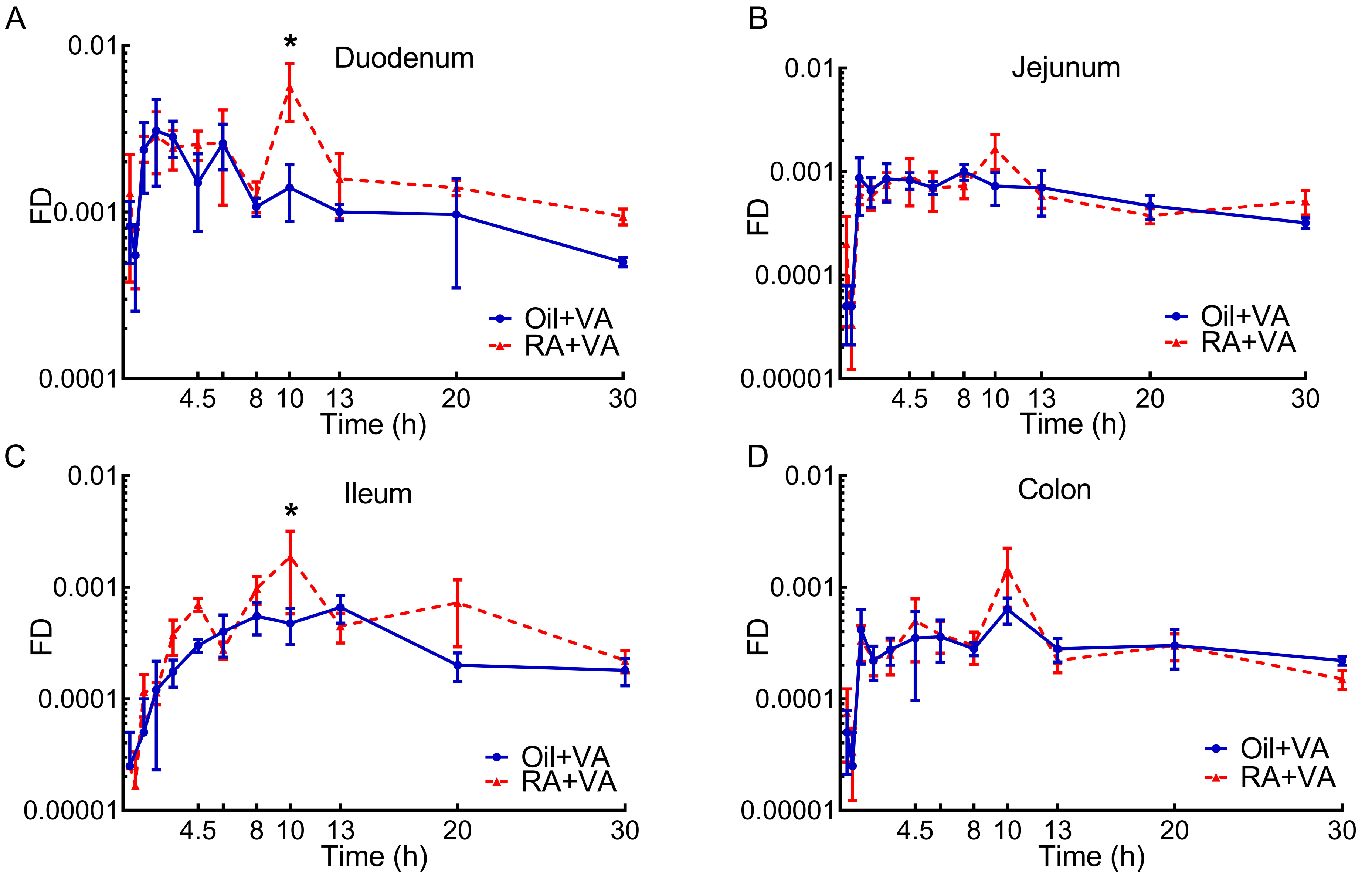

Figure S2. Fraction of ingested dose existing in retinol in different segments of intestine vs. time after administration of ^{3}H-labeled VA supplement in neonatal rats. (A)Duodenum, (B) Jejunum, (C) Ileum, (D) Colon. Data are presented as means ± SEM, *n*=4-6 pups/time/group. * indicates statistically significant difference between groups, $P < 0.05$.
